# A Sequential Vesicle Pool Model with a Single Release Sensor and a Ca^2+^-Dependent Priming Catalyst Effectively Explains Ca^2+^-Dependent Properties of Neurosecretion

**DOI:** 10.1371/journal.pcbi.1003362

**Published:** 2013-12-05

**Authors:** Alexander M. Walter, Paulo S. Pinheiro, Matthijs Verhage, Jakob B. Sørensen

**Affiliations:** 1 Department of Functional Genomics and Department of Clinical Genetics, Center for Neurogenomics and Cognitive Research, Neuroscience Campus Amsterdam, VU University Amsterdam and VU University Medical Center, Amsterdam, The Netherlands; 2 Department of Neuroscience and Pharmacology, Faculty of Health Sciences, University of Copenhagen, Copenhagen, Denmark; 3 Lundbeck Foundation Center for Biomembranes in Nanomedicine, University of Copenhagen, Copenhagen, Denmark; Research Center Jülich, Germany

## Abstract

Neurotransmitter release depends on the fusion of secretory vesicles with the plasma membrane and the release of their contents. The final fusion step displays higher-order Ca^2+^ dependence, but also upstream steps depend on Ca^2+^. After deletion of the Ca^2+^ sensor for fast release – synaptotagmin-1 – slower Ca^2+^-dependent release components persist. These findings have provoked working models involving parallel releasable vesicle pools (Parallel Pool Models, PPM) driven by alternative Ca^2+^ sensors for release, but no slow release sensor acting on a parallel vesicle pool has been identified. We here propose a Sequential Pool Model (SPM), assuming a novel Ca^2+^-dependent action: a Ca^2+^-dependent catalyst that accelerates both forward and reverse priming reactions. While both models account for fast fusion from the Readily-Releasable Pool (RRP) under control of synaptotagmin-1, the origins of slow release differ. In the SPM the slow release component is attributed to the Ca^2+^-dependent refilling of the RRP from a Non-Releasable upstream Pool (NRP), whereas the PPM attributes slow release to a separate slowly-releasable vesicle pool. Using numerical integration we compared model predictions to data from mouse chromaffin cells. Like the PPM, the SPM explains biphasic release, Ca^2+^-dependence and pool sizes in mouse chromaffin cells. In addition, the SPM accounts for the rapid recovery of the fast component after strong stimulation, where the PPM fails. The SPM also predicts the simultaneous changes in release rate and amplitude seen when mutating the SNARE-complex. Finally, it can account for the loss of fast- and the persistence of slow release in the synaptotagmin-1 knockout by assuming that the RRP is depleted, leading to slow and Ca^2+^-dependent fusion from the NRP. We conclude that the elusive ‘alternative Ca^2+^ sensor’ for slow release might be the upstream priming catalyst, and that a sequential model effectively explains Ca^2+^-dependent properties of secretion without assuming parallel pools or sensors.

## Author Summary

The release of neurotransmitter involves the rapid Ca^2+^-dependent fusion of vesicles with the plasma membrane. Kinetic heterogeneity is ubiquitous in secretory systems, with fast phases of release on the millisecond time scale being followed by slower phases. In the absence of synaptotagmin-1 – the Ca^2+^sensor for fast fusion – the fast phase of release is absent, while slower phases remain. To account for this, mathematical models incorporated several releasable vesicle pools with separate Ca^2+^ sensors. However, there is no clear evidence for parallel release pathways. We suggest a sequential model for Ca^2+^-dependent neurotransmitter release in adrenal chromaffin cells. We assume only a single releasable vesicle pool, and a Ca^2+^-dependent catalytic refilling process from a limited upstream vesicle pool. This model can produce kinetic heterogeneity and does better than the previous Parallel Pool Model in predicting the Ca^2+^-dependence of releasable pool refilling and the consequences of SNARE-protein mutation. It further accounts for the release in the absence of synaptotagmin-1 by assuming that the releasable vesicle pool is depleted, leading to slow and Ca^2+^-dependent fusion from the upstream pool, but through the same release pathway. Thus, we suggest that the elusive ‘alternative Ca^2+^ sensor’ is an upstream priming protein, rather than a parallel Ca^2+^ sensor.

## Introduction

Neurotransmitter release and synaptic transmission depend on the fusion of secretory vesicles with the plasma membrane by exocytosis, and the ensuing release of the contained neurotransmitter molecules. Exocytosis itself is the conclusion of a number of steps, which starts by the generation of the vesicle and its filling with neurotransmitter, and continues with the transport of the vesicle to the plasma membrane, its physical attachment to the membrane (docking), the attainment of fusion competence (priming), and ends with its fusion as the result of the arrival of a Ca^2+^ signal. The essential nature of Ca^2+^ for the final step of neurotransmitter release has been known since the pioneering work of Bernard Katz [1], whereas the high Ca^2+^-cooperativity of this step was demonstrated by Dodge and Rahamimoff [2]. Ca^2+^ uncaging made it possible to describe this cooperativity quantitatively and to derive mathematical models for the Ca^2+^ triggering step [3]–[6]. Later studies showed that at least one upstream replenishment step, probably vesicle priming, is also Ca^2+^-dependent [7]–[9] (for a review, see [10]). However, the arrangement of the different Ca^2+^-dependent steps with respect to each other is currently less than clear.

Initially, most working models assumed Ca^2+^-dependent vesicle priming and neurotransmitter release through a sequential pathway with one release sensor [11], [12]. Later on, to account for the observation of kinetically distinct (i.e. fast and slow) release phases, models incorporated different releasable vesicle populations, or pools [13]–[16]. These pools deviated from each other either in terms of molecular composition or localization with respect to Ca^2+^ channels. The notion of parallel releasable vesicle pools was reinforced when deletion of the Ca^2+^ sensor synaptotagmin (syt) (-1 or -2) was found to eliminate fast release, while Ca^2+^-dependent slow release components remained [17]–[19]. This led to the suggestion of multiple sensors in parallel either controlling a single [19], [20] or different vesicle pools [17]: in the absence of syt, a second release sensor would drive fusion.

Despite the fact that parallel pathways have been the working model for more than a decade, molecular correlates of the slow release pathway are still missing: syt-1, syt-2 and syt-9 are now widely accepted to be Ca^2+^ sensors for fast release [21], and detailed roles for many other proteins in fast release have been identified *in vivo* and reconstituted *in vitro*, including SNAREs, Munc13/CAPS and Munc18 [22], but no similar set of proteins dedicated to slow release is known (but see Discussion). Therefore, it remains important to consider alternatives and even return to sequential models to investigate if, with novel assumptions, such models suffice to explain the Ca^2+^-dependent properties of regulated secretion.

Based on properties derived from molecular perturbation studies, we propose a sequential model, where we make novel assumptions regarding the nature of the Ca^2+^-dependent priming steps. Recent studies concluded that initial assembly of SNARE-complexes is catalyzed by Ca^2+^-sensitive molecules such as Munc13 [23], [24] and coincides with priming [25]. Even though the word ‘catalyzed’ is often used loosely, we here decided to explore a model with a Ca^2+^-dependent catalytic mechanism in the strict sense, i.e. a mechanism, which increases both forward and backward rates by reducing the energy level of the transition state. In addition, to account for data obtained in syt-1 knockout cells (see below), we suggest that the Ca^2+^ sensor for fast release ensures the steady-state population of the Readily Releasable Pool (the RRP) at rest, either by preventing SNARE-dependent fusion in the absence and synchronizing fusion in the presence of Ca^2+^ [26]–[30], or by lowering the free energy of the RRP in the Ca^2+^-unbound state [31].

By mathematical modeling and comparison to older and new data we here show that these assumptions suffice to explain the Ca^2+^-dependence of secretion from chromaffin cells. Moreover, our model can account for recovery of the RRP after strong stimulation and deliver parsimonious explanations for the observed effects of SNARE or syt mutations without the need to evoke additional parallel pathways or release sensors. We suggest that a fresh look at sequential models is of value to understand kinetic diversity in secretory systems.

## Results

### Sequential and parallel vesicle pool models

**Figure 1:**
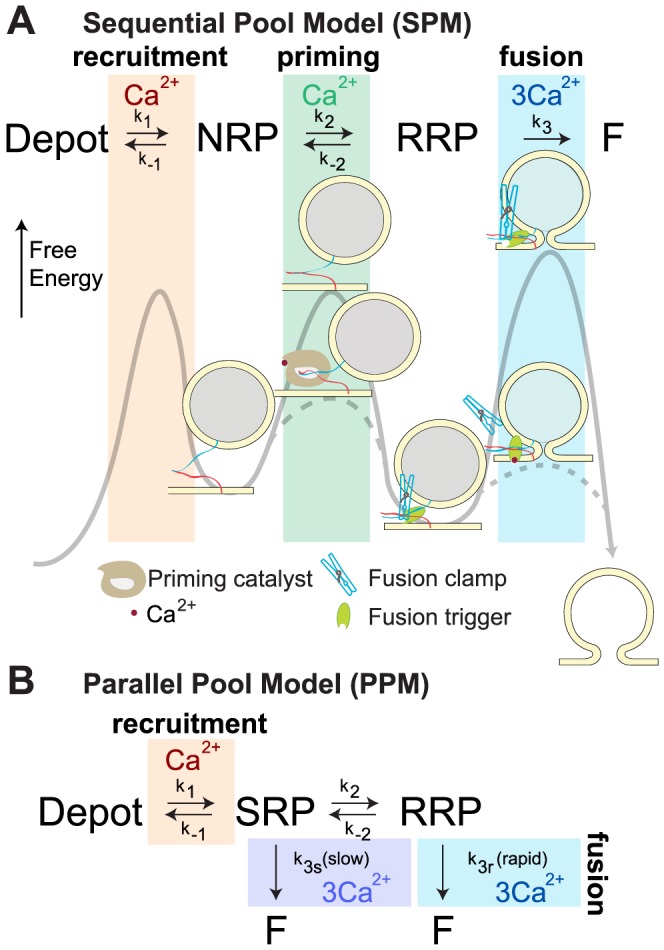
Two different models of secretion. A. Sequential Pool Model (SPM). The Depot, Non-Releasable Pool (NRP), Readily-Releasable Pool (RRP) and Fused (F) states are represented by local minima of the free energy. Reactions between these states require the crossing of transition states that represent local maxima of the energy landscape. A Michaelis-Menten Ca^2+^-dependent step fills the NRP from the Depot Pool (recruitment, brown background). A Ca^2+^-dependent catalyst decreases the energy of the transition state for priming (transition to the RRP state) in a Ca^2+^-dependent manner, thus increasing the rates of both forward and backward reactions (priming, green background). A downstream clamp arrests release by imposing a high-energy transition state for fusion, which can be removed by Ca^2+^ binding to the associated sensor, thereby enabling fusion (fusion, blue background). The details of the cooperative Ca^2+^ sensor for fusion is given in Fig. 2A. **B. Parallel Pool Model (PPM).** In this model, the same Michaelis-Menten Ca^2+^-dependent step fills the SRP from the Depot Pool, as in the SPM (recruitment, brown background). Both the Slowly-Releasable Pool (SRP) and the Readily-Releasable Pool (RRP) are releasable, with different cooperative Ca^2+^ sensors (fusion, dark and light blue background, shown in detail in Fig. 2B). The transition between SRP and RRP is Ca^2+^-independent (after [16] ).

**Figure 2:**
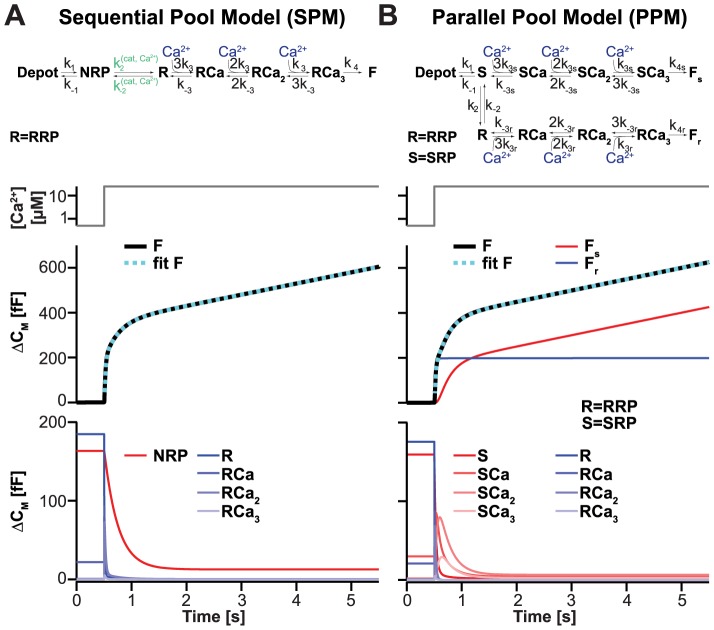
Both the SPM and the PPM predict a biphasic burst of release. **A.** Top panel: Full Sequential Pool Model (SPM), including details of the cooperative Ca^2+^ sensor. Abbreviation: R = RRP. Lower panels: The model was solved in the steady-state using parameters in Table 1, and simulated over time with a step increase in [Ca^2+^]_i_ from 0.5 to 25 µM at 0.5 s (grey lines). The development of the pools (bottom panel) shows fast depletion of all RRP-states (blue), concomitant with the fastest phase of burst release (middle panel). The NRP (red) is depleted slower and only partially, giving rise to the slower phase of burst release. During the subsequent sustained component the recruitment of vesicles from the Depot, followed by maturation through the NRP and RRP states, support ongoing release. The dotted blue line in the middle panel is a fit of a sum of two exponential functions plus a straight line to the model simulation. **B.** Top panel: Full Parallel Pool Model (PPM), including details of the fast and slow cooperative Ca^2+^ sensors. Abbreviations: R = RRP; S = SRP. Lower panels: The model was solved in the steady-state using parameters in Table 2, and simulated over time with a step increase in [Ca^2+^]_i_ from 0.5 to 25 µM at 0.5 s. The development of the pools (bottom panel) shows fast depletion of all RRP-states (blue), concomitant with the fastest phase of burst release (middle panel). The SRP is depleted slower, giving rise to the slower phase of burst release. During the subsequent sustained component recruitment of vesicles from the Depot to the SRP is followed by fusion through the slow Ca^2+^ sensor, without maturation to the RRP state (middle panel: F_s_ is fusion through the slow pathway, F_r_ is fusion through the fast pathway). The dotted blue line in the middle panel is a fit of a sum of two exponential functions plus a straight line to the model simulation.

In order to explain the complex Ca^2+^-dependent properties of regulated exocytosis, we investigated a mathematical model with sequentially arranged pools (Sequential Pool Model, SPM), separated by Ca^2+^-dependent steps [11] (Fig. 1A). Our model assumes that some vesicles reside in a Non-Releasable Pool (NRP) from which they can undergo reversible priming to a Readily-Releasable Pool (RRP). From there, vesicles can irreversibly converge to the fused state (F). Vesicles enter the NRP by recruitment from a much larger Depot Pool (Fig. 1A). This recruitment is Ca^2+^-dependent, driven by a Michaelis-Menten Ca^2+^-dependent forward process (k_1_) with a K_d_ of 2.3 µM, as in previous models of the adrenal chromaffin cell [16], [32]. At rest, the supply of vesicles to the RRP from the NRP can be slow, because the demand is low. However, during repetitive stimulation, RRP vesicles may soon be depleted unless Ca^2+^ speeds up their re-supply, as observed experimentally [9], [32]. Here, we investigated the possibility that the process that enhances priming (i.e. transition from NRP to RRP) is Ca^2+^-dependent catalysis. A catalyst lowers the activation energy barrier of a reaction (Fig. 1A), thereby simultaneously increasing both forward (k_2_) and backward (k_−2_) rates (see Materials and Methods). Thus, unlike the selective increase of the forward rate, a catalyst speeds the transition without affecting the proportion of NRP- to RRP-vesicles in equilibrium. This complies with data in the adrenal chromaffin cells, as shown below. The Ca^2+^ trigger for fusion is modeled as a sequential sensor binding 3 Ca^2+^ ions, followed by a Ca^2+^-independent, but very fast, conversion to the Fused state [16] (rate constant k_4_, not shown in Fig. 1A; see Fig. 2A). In thermodynamic terms, the Ca^2+^ trigger affects the energy barrier for fusion. This barrier is high in the absence and low in the presence of Ca^2+^, resulting in strongly Ca^2+^-dependent fusion rates (Fig. 1A).

We modeled release from chromaffin cells using the SPM to investigate whether previously obtained experimental data could be explained by the sequential Ca^2+^-dependent actions we suggest. For comparison, we simulated the Parallel Pool Model (PPM), where this had not already been done in the literature [16], [32] (Fig. 1B). In the PPM, parallel fusion of two releasable pools, the Slowly-Releasable Pool (SRP) and the (fast) Readily-Releasable Pool (RRP), is assumed. The Ca^2+^ sensors for RRP and SRP fusion are separate sequential and cooperative sensors each binding three Ca^2+^ ions (see Materials and Methods). Like in the SPM, supply of vesicles from the larger Depot Pool is a Ca^2+^-dependent Michaelis-Menten process [16]. The parameters of this step as well as the ones for the Ca^2+^-binding and fusion from the RRP were identical in the two models. Each model was solved in the steady-state at the beginning of each simulation, and then allowed to evolve by numerical integration, driven by the Ca^2+^ signal characteristic for each stimulation protocol. The cumulative release was calculated, from which release rates and pool sizes were determined by fitting a sum of exponential functions. Parameters were taken or estimated from published data, as described in Materials and Methods, and are listed in Table 1 (SPM) and Table 2 (PPM), respectively.

**Table 1:** SPM model parameters for chromaffin cells.

Parameter	Value	comment
*k_1_*	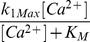	[16]
*k_1Max_*	55 fF/s	[16]
*K_M_*	2.3 µM	[16]
*k_−1_*	0.05 s^−1^	[16]
*n*	1	cooperativity catalyst
*k_2_*	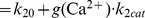	see Materials and Methods
*k_−2_*	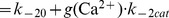	see Materials and Methods
*g(Ca^2+^)*	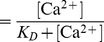	see Materials and Methods
*k_20_*	0.021 s^−1^	see Materials and Methods
*k_2cat_*	20 s^−1^	see Materials and Methods
*k_−20_*	0.017 s^−1^	see Materials and Methods
*k_−2cat_*	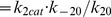	see Materials and Methods
*K_D_*	100 µM	see Materials and Methods
*k_3_*	4.4 s^−1^µM^−1^	[16]
*k_−3_*	56 s^−1^	[16]
*k_4_*	1450 s^−1^	[16]

**Table 2:** PPM model parameters for chromaffin cells.

Parameter	Value	comment
k1	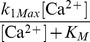	[16]
*k_1Max_*	55 fF/s	[16]
*K_M_*	2.3 µM	[16]
*k_−1_*	0.05 s^−1^	[16]
*k_2_*	0.12 s^−1^	[16]
*k_−2_*	0.1 s^−1^	[16]
*k_3s_*	0.5 s^−1^µM^−1^	[16]
*k_−3s_*	4 s^−1^	[16]
*k_4s_*	20 s^−1^	[16]
*k_3r_*	4.4 s^−1^µM^−1^	[16]
*k_−3r_*	56 s^−1^	[16]
*k_4r_*	1450 s^−1^	[16]

### Ca^2+^ uncaging experiments and biphasic release

Pioneering work [6], [16], [32] using Ca^2+^ uncaging and cellular capacitance recordings resulted in the identification of fast and slow release components in adrenal chromaffin cells. After parameter estimation, we simulated both models (SPM, PPM) using an abrupt Ca^2+^-step from 0.5 µM to 25 µM (Fig. 2). Both models displayed biphasic capacitance responses, in agreement with published data. The fastest phase is referred to as the ‘fast burst’ of release and has a rate of ∼50 s^−1^ under these conditions. The subsequent slow burst of release is approximately 10-fold slower. Following these two components, release persists in the so-called sustained phase, which is nearly linear. In the ‘classical’ PPM, the fast burst component is caused by the fusion of vesicles from the RRP, whereas the slow burst component owes itself to the fusion of SRP-vesicles (Fig. 2B). Finally, when both RRP and SRP have been depleted, newly recruited vesicles (from the Depot Pool) fuse continuously as long as the intracellular Ca^2+^ concentration ([Ca^2+^]_i_) remains high, giving rise to the sustained component of release. A particularity of the PPM is that fusion during the sustained phase is almost entirely through the SRP, since the SRP-to-RRP conversion is relatively slow and Ca^2+^-independent (Fig. 2B, middle panel: F_s_ gives the fusion through the slow pathway, F_r_ gives the fusion through the fast (rapid) pathway) [33].

In the sequential model (SPM), the fast burst component corresponds mainly to the rapid depletion of RRP-vesicles, whereas the slow burst component corresponds mainly to NRP-vesicles that mature to the RRP-state before fusing (Fig. 2A). This maturation step is slower than fusion from the RRP and it is Ca^2+^-dependent, driven by the Ca^2+^-dependent catalyst, giving rise to the slow burst phase. It should be noted that the identification of the fast component with the RRP and the slow component with the NRP is only approximate, because the size and kinetics of the two phases depend on the entire system (Materials and Methods). Therefore, to identify fast and slow burst components we fitted simulated traces with a sum of exponentials in order to compare these values to the ones obtained from experiments in the same way. Finally, when both the NRP and RRP are empty, sustained release is driven by the upstream Ca^2+^-dependent reaction from the Depot Pool (k_1_, Fig. 1). Other than in the PPM, these vesicles also fuse through the RRP, which is the only releasable state in the SPM.

Since both models could reproduce biphasic burst release and the sustained component, we next compared our new model with data that had previously been fitted with the PPM [16].

### Vesicle pools and fusion kinetics

**Figure 3:**
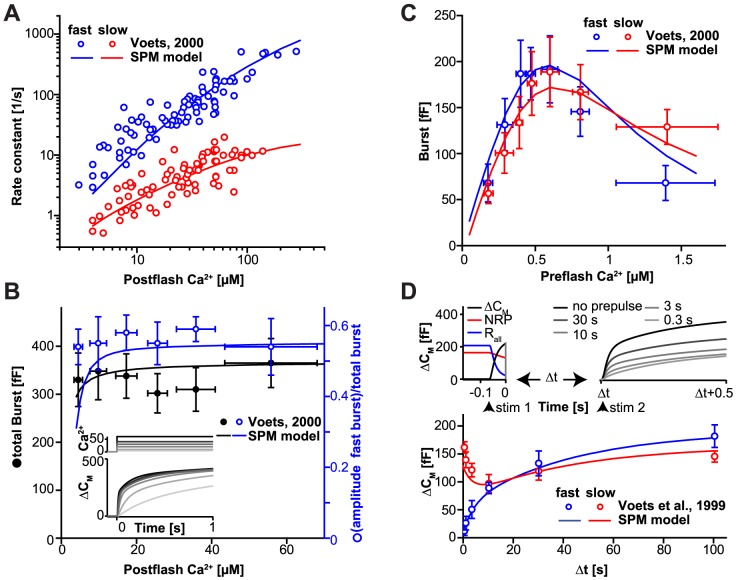
The SPM fits experimental data from chromaffin cells. **A.** The fast (blue) and slow (red) fusion rates are sensitive to the Ca^2+^ levels reached after Ca^2+^-uncaging (data points from [16], and model predictions of the SPM in solid lines). **B.** In contrast, the sum of the fast and slow burst component ( = total burst, black, left hand side), or the fraction of fast- to total burst release (blue, right hand side) are fairly insensitive to post-flash Ca^2+^ (data points, from [16], and model predictions of the SPM in solid lines). The inset shows examples of model simulations (top panel, Ca^2+^ signal; bottom panel, the calculated capacitance responses). **C.** The amplitudes of the fast (blue) and slow (red) burst components are a bell-shaped function of the resting Ca^2+^ levels (data points from [16] and model predictions in solid lines). The increase at low [Ca^2+^]_i_ is due to the Ca^2+^-dependent recruitment from the Depot Pool, whereas the decrease at higher [Ca^2+^]_i_ is due to partial pool depletion. **D.** Bottom panel: following selective depletion of the fast component, its recovery is slow and at the cost of the slow component, in agreement with experiments (data points from [32] and model predictions of the SPM in solid lines). The top panel illustrates our simulation paradigm: a stimulation that selectively depleted the R-state (the major determinant of the fast component) was followed by a second stimulation that depleted the total releasable pool at varying intervals (Δt). (See Table 1 for parameter values).

Systematic variation of the Ca^2+^ signal driving the release model allowed the determination of amplitudes and rate constants of fast and slow burst components as a function of pre- and post-flash Ca^2+^ concentrations (data points in Fig. 3A, 3B, and 3C, from [16]). Experimentally it was found that the time constants depended on the Ca^2+^ levels reached after Ca^2+^ uncaging (Fig. 3A), whereas the number of vesicles released in each phase was relatively independent of post-flash [Ca^2+^]_i_ (data points in Fig. 3B, from [32]). Our model accounts well for the kinetic data (model simulations are shown as lines in Fig. 3; for examples of simulated capacitance traces see insert in Fig. 3B). In further agreement with the SPM, both slow and fast burst components were augmented with moderate increases of resting (pre-flash) [Ca^2+^]_i_ below 0.7 µM, owing to the Ca^2+^-sensitive supply rate (k_1_ in Fig. 1) from the Depot (Fig. 3C, model simulation are lines, data are points), while at higher pre-flash [Ca^2+^]_i_ the size of both components decreased, due to the partial depletion of the underlying pools (NRP, RRP) through release before the uncaging event. However, the relative proportion of fast and slow components remained fairly constant (Fig. 3C). The invariance in fast-to-slow amplitude when changing the Ca^2+^ concentration originally served as an argument for a direct slow fusion pathway in the PPM, since it seemed inconsistent with a Ca^2+^-dependent interconversion between fast and slow vesicles. However, we show here that when modeling Ca^2+^-dependent priming as a catalytic process, the invariance is maintained in a sequential model (lines in Figs. 3C and 3B), except for very low post-flash Ca^2+^ concentrations (Fig. 3B), where the distinction between fast and slow burst components becomes experimentally challenging.

Another stimulation paradigm probed the recovery of the fast burst component in chromaffin cells [32]. The stimulation paradigm combined depolarizations and Ca^2+^ uncaging to allow a selective depletion of the fast component without affecting the slow component. It was found that the recovery of the fast component occurred at the loss of the slow component (data points in Fig. 3D, from [32]). This behavior is also an inherent feature of our sequential model: the refilling of fast (RRP) vesicles after selective depletion occurred at the cost of NRP vesicles, which are the major source of the slow burst (lines in Fig. 3D).

In the above, we simulated a model where only a single Ca^2+^ bound to the priming catalyst (cooperativity one). We also constructed a version of the model with a cooperativity of two (Materials and Methods). Fitting this model to the data also resulted in a satisfactory fit (Fig. S1, Table S1). The fit to the fast and slow burst components as a function of preflash [Ca^2+^]_i_ was a little better (Fig. S1C), but the fit to the fast burst fraction as a function of postflash [Ca^2+^]_i_ was a little worse (Fig. S1B). Thus, the data do not allow a clear conclusion as to whether the catalyst has a cooperativity of one or two. Therefore, we continued exploring the simplest model with cooperativity one.

We conclude that the now classical data of Thomas Voets on fast and slow release phases and fusion kinetics in mouse chromaffin cells can all be satisfactorily fit by our sequential model (SPM), as well – as previously shown [16] – by the PPM.

### Recovery of the RRP after strong stimulation

One fundamental difference between the PPM and the SPM is the recovery behavior of the RRP after its depletion. In the PPM, the refilling of the RRP from the SRP is Ca^2+^-independent. This Ca^2+^-independence was concluded from the observation of parallel enhancement of the fast and slow burst by increasing steady state [Ca^2+^]_i_ in the sub-micromolar range before stimulation [16] (Fig. 3C). However, as shown above, such a behavior is also inherent to the SPM, as long as the Ca^2+^-dependent acceleration of priming is catalytic. Therefore, the two models can be distinguished by probing the recovery of the RRP under conditions of elevated Ca^2+^: according to the SPM, its recovery should be sped up, whereas the PPM predicts Ca^2+^-independent - and thus slower - recovery.

**Figure 4:**
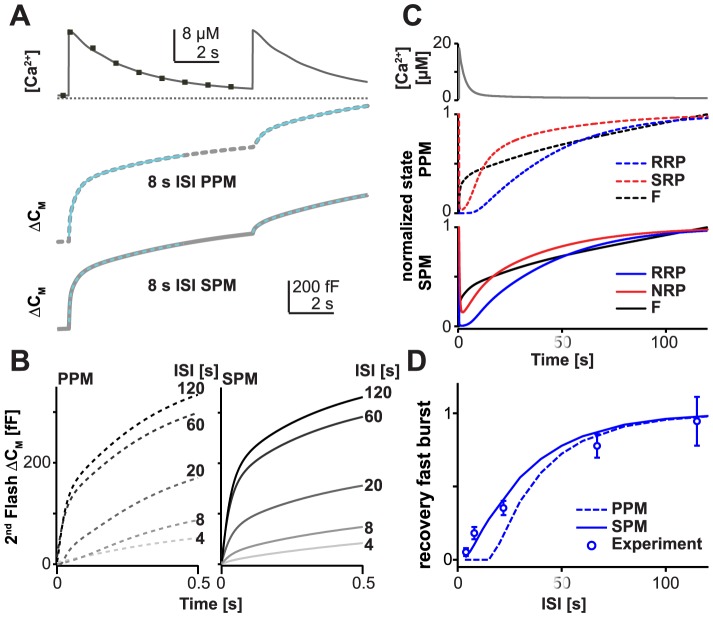
The SPM explains recovery of the fast component after strong stimulation. **A.** Top panel: double Ca^2+^ uncaging protocol (points, data; lines, Ca^2+^ signal used to drive the models). Lower panels: simulated capacitance traces for the two models (PPM and SPM, Figure 1) during the double uncaging protocol with Inter-Stimulus Interval (ISI) 8 s. **B.** Capacitance changes elicited by the 2^nd^ uncaging event after different ISIs and simulated by both models (left panel, PPM; right panel, SPM). **C.** Development of the pools over time in the two models (PPM and SPM), following the first uncaging event. For the PPM, the SRP and RRP vesicles not bound to Ca^2+^ are shown. For the SPM, the RRP not bound to Ca^2+^ is shown. It is noticeable that recovery of the RRP is delayed in the PPM compared to the SPM. **D.** Recovery of the fast burst component (identified by fitting of exponentials) in the experiment (points: experimental data from [34]), and as predicted by the two models (PPM: dotted line; SPM: full line). Only the SPM predicts the fast recovery of the fast component seen experimentally.

Recently, we used a dual-uncaging protocol to investigate the recovery of the RRP after Ca^2+^-uncaging had emptied both the fast and the slow burst components [34]. Applying a second Ca^2+^ uncaging flash at variable inter-stimulus intervals (ISI) while measuring Ca^2+^-relaxation (Fig. 4A, top panel) allowed estimating the RRP recovery (points in Fig. 4D). Note the difference in approach to the previous selective depletion of the RRP under conditions where Ca^2+^ presumably relaxed to baseline much faster (Fig. 3D). We found that already at 8 s the fast component had recovered to 18.1±4.2% of the initial value, and at 22 s recovery was at 35.3±4.9% (symbols in Fig. 4D, note that all lines in Fig. 4 represent simulations, not data). This behavior is inconsistent with the PPM (broken line in Fig. 4D), which featured a full ∼10 s delay, before the first recovery of the fast component was visible, owing to the lack of Ca^2+^-accelerated refilling of the RRP. This is also clearly appreciated from the simulations of the capacitance increases (Fig. 4A, B), which lack a fast component in the PPM at these time points. Looking at the evolution of the pools in the PPM (Fig. 4C) it is clear that the RRP does not start to recover appreciably within the first 10 s. In contrast, the SPM in fact predicted the faster recovery of the fast component without the need to adjust parameters (i.e. the same parameters were used here as in Fig. 2 and 3) (Fig. 4D). On the simulation of single traces, the fast burst is clearly seen at short interstimulus intervals (Fig. 4A, B). In spite of the quicker recovery of the fast component in the SMP, its recovery still lagged behind the recovery of the slow component (Fig. 4B and Fig. S2). The faster recovery of the slow pool is a fundamental feature of neurotransmission also found in the Calyx of Held synapse [35].

The faster recovery in the SPM is a potentially physiologically important property, which allows secretory cells to regain potency for fast release quickly after stimulation. Clearly, the SPM is more consistent with this type of experiment. The recovery ‘pause’ in the PPM due to the Ca^2+^-independent SRP-to-RRP conversion is a fundamental feature of that model and it would therefore not be possible to account for this behavior while still accounting for the data in Fig. 3 by mere adjustment of the model's parameters. We therefore sought to modify the PPM in such a way that this important property could be reproduced by a model with parallel fusion pathways by speeding the recovery of the RRP in a Ca^2+^-dependent manner. As described above, this cannot be achieved by an increase in the SRP-to-RRP forward conversion rate alone, as this would change steady-state pool sizes (inconsistent with Fig. 3). Therefore we made use of our idea of a Ca^2+^-dependent catalyst, which is used in the SPM, and incorporated catalysis in the SRP-to-RRP inter-conversion, so that once again forward and reverse rates were Ca^2+^-dependent (Fig. S3A). Indeed, such a model (PPM+cat) was also able to account for the general Ca^2+^-dependence of release (Fig. S3A–C, Table S2) while the catalysis of the SRP-RRP inter-conversion sped the recovery of the fast component at elevated Ca^2+^ to a closer agreement with the experimental data (Fig. S3D).

In conclusion, the classical PPM failed in reproducing the faster recovery of the fast component after strong stimulation, while the SPM or the modified PPM+cat, which both feature Ca^2+^-dependent catalysis in RRP replenishment, matched the data more closely. In our opinion this still argues for the SPM rather than the PPM+cat, because the SPM is simpler, has fewer parameters and does not require a second fusion pathway, while it can account for all experiments investigated.

### Perturbation of the last fusion step affects release components and fusion rates

**Figure 5:**
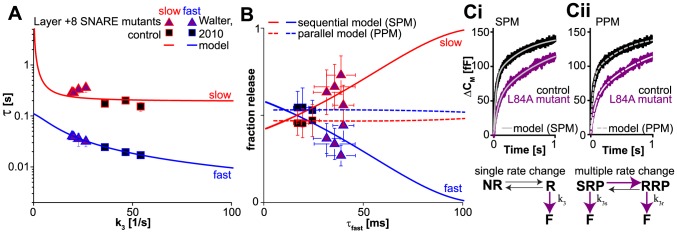
The SPM predicts the observed inter-dependence of release rates and amplitudes following regional SNARE mutation. (**A** and **B**) Evaluation of the SPM model in comparison to experimental data obtained after C-terminal SNARE mutation. **A.** According to the SPM model, the time constants of both fast (blue line) and slow burst release (red line) depend on the fusion rate *k_3_*. The data points (from [25]) show the measured time constants of fast (blue edges) and slow (red edges) release in wildtype (control, black squares) and synaptobrevin-2 mutants (Layer +8 mutants, purple triangles) bearing a mutation in layer +8 (residue 84: L to A, D, N or G). **B.** According to the SPM model, release changes from mainly fast (blue) to mainly slow (red) with increased values of the fast time constant. This is in line with experimental data from C-terminal SNARE mutants (purple triangles), which show an increase in the fast time constant of release as well as an increase in slow (red edges) and a decrease in fast (blue edges) burst release. This behavior cannot be accounted for by a single parameter change in the Parallel Pool Model (PPM, simulation, dashed lines). **C.** Comparison of the SPM and the PPM models. **Ci.** Top panel: the SPM model fits well to average experimental data [25] and accounts for differences between control (wildtype synaptobrevin-2, black) and mutant (an L84A mutant, purple) by a change of a single parameter (bottom panel). **Cii.** Top panel: the PPM fits well to average experimental data [25], but the effect of L84A mutation requires the adjustment of multiple parameters (white, unevenly dashed line and bottom panel). Model predictions in panel A and B were calculated with *k_3_* as independent variable, the relationship between *τ_fast_* and *k_3_* given in Methods and with values of *k_2_* and *k_−2_* determined by a global fit to the data in panels A and B (*k_2_* = 5.26 s^−1^ and *k_−2_* = 3.80 s^−1^). Error bars represent SEM.

Thus far, the SPM is in line with experimental data describing vesicle pools, fusion kinetics and RRP recovery. The recovery after strong stimulation particularly favors the SPM over the PPM. Another difference between the models is in the predicted effect of a change in the fast release rate. In the PPM, release rates and amplitudes are independent. Therefore, a change in any one of these parameters is expected to leave all others (almost) unaffected. In contrast, in the SPM model, the amplitudes and release rates are all functions of the elementary rate constants k_2_, k_−2_ and k_3_ (see Materials and Methods). Therefore, the time constants and amplitudes of fast and slow burst release are interdependent (due to their individual dependence on k_2_, k_−2_ and k_3_) and a change in k_3_ is expected to affect both fast and slow release. This is demonstrated in Fig. 5A where we have calculated the time constants of fast and slow burst release while varying the final fusion rate k_3_ (lines are model simulations). Moreover, the relative contribution of fast and slow release depends on the time constant of fast release (Fig. 5B), reducing k_3_ will therefore also change the ratio of fast to slow release.

It is commonly assumed that one of the last events before vesicle fusion is the assembly of the final interaction ‘layer’ (layer +8) in the SNARE complex. In the SPM this molecular event is primarily reflected by the rate constant k_3_ (although it might affect k_2_ as well). Mutagenesis studies of layer +8 in synaptobrevin-2 previously performed by us [25] indeed resulted in the identification of an increase in the fast time constant (data points in Fig. 5A). In the PPM one might expect that the release rates of both fast and slow vesicles are decreased if both fusion reactions required the C-terminal interaction of synaptobrevin-2. However, changes in the proportion of fast to slow burst release are not expected. In contrast to this we found that mutation of layer +8 resulted in a shift of the relative contribution of fast and slow release; as fast release became slower, the amplitude of the fast component decreased while the amplitude of the slow component increased ([25]; data points in Fig. 5B). With the SPM we can explain the connection between these effects in the framework of a simple model (solid lines in Fig. 5): C-terminal SNARE destabilization reduces k_3_ (illustrated by the purple arrow in Fig. 5Ci), resulting in an increased time constant for fast release (τ_fast_, Fig. 5A) and a simultaneous change in fast and slow contribution to release (Fig. 5B). The PPM cannot account for the simultaneous change of release rates and amplitudes by a single parameter change, because pool sizes and release rates are independent. Therefore, changes in the fast release rate are predicted to leave the relative contribution of fast and slow release unchanged (dashed lines in Fig. 5B are simulations of the PPM), which is not easily reconciled with the experiment. The PPM can still mathematically describe the data, but for this it is necessary to assume that several rates are changed by the single point mutation, including one that changes the relative sizes of the RRP and SRP (purple arrows in Fig. 5Cii). Hence, while both models fit the data, the SPM delivers a straightforward explanation for the observed relationship of fast release rates and amplitudes in general and relates the main effect of C-terminal SNARE mutation to a single rate constant (k_3_) in particular.

Thus, a sequential model featuring an upstream Ca^2+^-dependent catalyst with a downstream Ca^2+^ sensor predicts the effects of SNARE mutations.

### Deletion of synaptotagmin-1: Unclamping release, or destabilizing the RRP?

One argument for parallel pool or parallel sensor models has been the observation that after deletion of the fast Ca^2+^ sensor in various systems slow release components persist. This is also the case in mouse adrenal chromaffin cells, where deletion of syt-1 leads to ablation of the fast burst component, whereas the slow burst component and the sustained component both remain [17], [31], [36]. Clearly, a Parallel Pool Model, where the RRP would utilize syt-1 and the SRP a separate Ca^2+^ sensor (even though no such molecule has been found yet) is an easy and reasonable way to account for this finding. How can one account for it in a sequential model?

**Figure 6:**
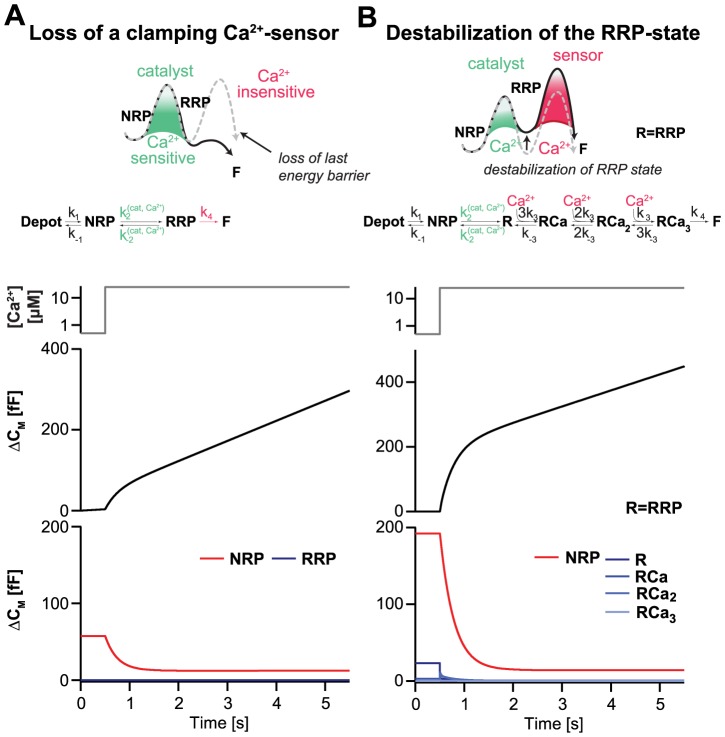
Destabilizing or spontaneously fusing the RRP in the SPM qualitatively mimics the syt-1 knockout phenotype. **A.** In the first version of the model simulating syt-1 null data, we assumed that the last barrier to fusion is gone (top panel, grey dotted line shows the energy landscape in the WT case), and the RRP vesicles fuse independently of Ca^2+^ with the maximal rate constant, *k_4_* (middle panel, compare to Fig. 2A). The SPM was solved in the steady-state, and simulated over time with a step increase in [Ca^2+^]_i_ from 0.5 to 25 µM at 0.5 s. The capacitance increase displays a slow and a sustained component, but no fast component (bottom panels, compare to Fig. 2A), in qualitative agreement with experimental data (see text). Bottom panel: the temporal development of pools shows that the RRP-state was depleted even before the Ca^2+^ step. The NRP was also partially depleted, leading to a smaller slow component than in the wildtype case (compare to Fig. 2A). **B.** In the second version of the model simulating syt-1 null data, we assumed that the RRP would be destabilized and thus we increased the *k_−2_* by a factor of 10 (top panel, grey dotted line shows the energy landscape in the WT case). Simulating a step increase in [Ca^2+^]_i_ from 0.5 to 25 µM revealed a large slow burst of release, whereas the fast burst is small and hardly noticeable. The NRP size is even a bit larger than in the WT case (bottom panel, compare to Fig. 2A). Note that, in both versions of the syt-1 null, it is the Ca^2+^-dependent catalyst between the NRP and the RRP which drives secretion and determines its Ca^2+^ sensitivity.

Recent *in vitro* and *in vivo* experiments have emphasized the function of syts and/or complexins as clamps on release [26]–[30]. Therefore, one idea is that these proteins – probably working in concert – are able to arrest SNARE complex assembly. Ca^2+^-binding to syt would release the clamp and allow the SNARE complex to complete its assembly, leading to rapid membrane fusion. In our model, the clamp would generate the final energy barrier for fusion (Fig. 1A), which could be removed by the binding of three Ca^2+^ ions to syt-1 (Fig. 2A). In this scenario one can model the syt-1 null data by removing the final fusion barrier, making RRP vesicles fuse with the maximal rate (k_4_) (Fig. 6A, top panel). This fast – and now Ca^2+^-independent – fusion from the RRP leads to chronic pool depletion. As a result of the missing clamp, the NRP vesicles in effect become releasable, with the transition from NRP to RRP being rate limiting for fusion. This transition is sped up by the Ca^2+^-dependent catalyst (Fig. 1), resulting in Ca^2+^-dependent, but much slower, release rates. We simulated our model under these conditions driven by a Ca^2+^ uncaging event (Fig. 6A, bottom panels). Indeed, our model predicted a missing fast component of release, and the persistence of both slow and sustained release components [17], [31], [36]. The slow component was driven by the sequential transition of vesicles from the NRP to the RRP to the F-state, following an increase in [Ca^2+^]_i_. The slow component of release was somewhat smaller in the syt-1 null than in the WT case, because of partial depletion of the NRP at rest (comp. Fig. 6A and Fig. 2A). Because of the lack of the last energy barrier, ongoing release at rest depleted the RRP (and the NRP partially) and led to an increase in the spontaneous release rate from 1.7 fF/s to 6.9 fF/s.

Another way of accounting for the selective loss of the fast burst component in the SPM model is to assume that syt-1 stabilizes the RRP state, by lowering its free energy. This is in line with previous data showing that syt-1 overexpression increases the RRP/SRP ratio [31]. We simulated this by increasing k_−2_ by a factor of 10 (Fig. 6B). Thermodynamically, this corresponds to increasing the energy state of the RRP, plus the subsequent energy barrier. Simulating this situation resulted in only a very small fast burst, followed by a slow burst and a sustained component (Fig. 6B). Under these circumstances, the NRP was a bit larger than in the WT case (Fig. 2A), leading to a slightly larger slow burst. In this version of the model, the properties of the Ca^2+^ triggering step remained unchanged. This might appear unrealistic for a simulation of the syt-1 null, because syt-1 is usually supposed to provide the Ca^2+^ binding sites. However, since most vesicles are present in the NRP, and the NRP-to-RRP transition is rate limiting for the majority of release, the properties of the Ca^2+^ sensor can in fact be changed simultaneously without noticeably changing the kinetics of overall release. In other words, when the majority of vesicles are present in the NRP, the model is pretty insensitive to the details of the RRP-to-F conversion.

We conclude that the SPM offers at least two possibilities for qualitatively accounting for the selective loss of the fast burst component, as observed in the syt-1 null, without assuming parallel release sensors or vesicle pools: either by assuming that the RRP is emptied because of spontaneous fusion, or by assuming that the RRP is destabilized, so that most vesicles reside in the NRP instead.

## Discussion

The idea of parallel organized readily-releasable and slowly-releasable vesicle pools with separate release pathways has been influential. Here, we showed that a sequential model with a few new assumptions does as well as parallel models in describing the size and kinetics of release phases in chromaffin cells, as probed by Ca^2+^-uncaging experiments (Figs. 2–3). The model is better at accounting for fast recovery after strong stimulation (Fig. 4), and it delivers parsimonious explanations for observations obtained with C-terminal synaptobrevin mutations (Fig. 5). We also showed that by assuming either 1) that syt-1 clamps release from the RRP, or 2) that syt-1 stabilizes vesicles in the RRP the sequential model could account for the loss of fast burst secretion in the syt-1 null cells (Fig. 6). This introduces testable interpretations of the remaining secretion in syt knockouts (see below).

### A Ca^2+^-dependent catalyst for priming

The Parallel Pool Model (PPM) assumed that Ca^2+^-dependent priming (refilling of the SRP) indirectly led to refilling of the RRP through a Ca^2+^-independent process (Fig. 1B). A Ca^2+^-independent SRP-to-RRP inter-conversion was assumed, because this could account for the parallel increase in SRP and RRP size with increasing Ca^2+^ concentrations at rest (Fig. 3C). If merely the forward rate were Ca^2+^-dependent, then the RRP-to-SRP ratio would change with [Ca^2+^]_i_, which was inconsistent with the experiment. However, the slow, Ca^2+^-independent RRP refilling reaction made it impossible to account for the slow burst of release by fusion through the RRP state alone. Instead, it was assumed that the SRP-vesicles could fuse directly through a separate pathway, which in turn necessitated a separate Ca^2+^ sensor for this pool. This arrangement seemed to be confirmed when deletion of syt-1 led to a specific deletion of the fast burst of release [17].

We solved the problem of the invariant slow-to-fast release ratio in a different way: by assuming that the NRP-to-RRP conversion is driven by a Ca^2+^-dependent catalyst. This in effect makes both the forward and the reverse reactions Ca^2+^-dependent. Moreover, because the catalyst acts on the transition state (Fig. 1A and Materials and Methods), it follows immediately that the Ca^2+^-dependence of forward and reverse rates are identical, ensuring the invariance of the NRP/RRP ratio. The catalytic process then speeds up refilling of the RRP to the degree that fusing the upstream NRP vesicles after transit through the RRP accounts for the slow burst of release in uncaging experiments, while this transition is slow at rest. This removes the need to assume a separate release pathway for those vesicles. Thus, in our model the ‘slow’ vesicles are no longer releasable, which is why we renamed this state Non-Releasable (NRP). Moreover, the Ca^2+^-dependent NRP-to-RRP conversion speeds up RRP refilling, which makes it possible to explain the faster recovery kinetics upon strong stimulation (Fig. 4) that could not be explained in the PPM. Thus, modeling the Ca^2+^-dependence of priming as a catalytic process explains a range of phenomena. Ca^2+^-dependent refilling of the RRP is a physiologically important mechanism, which ensures that the same signal that causes release from the RRP also speeds up refilling. We also managed to modify the PPM by the incorporation of a Ca^2+^-dependent, catalytic acceleration of RRP refilling (PPM+cat, Fig. S3), which could account for the data in a parallel model with additional parameters. This emphasizes the advantage of modeling RRP replenishment as a catalytic process independent of whether release commences in parallel or not. Moreover, catalysis accelerates priming under high-use conditions while strictly preventing ‘overfilling’ of the RRP, which is characteristic of models with non-catalytic Ca^2+^-dependent priming [12].

Since vesicle priming coincides with SNARE-complex formation [25], [37], the molecular counterpart of the catalyst should be sought amongst Ca^2+^-dependent proteins which have been shown to facilitate SNARE-complex assembly; likely candidates are Munc18, Munc13 and CAPS proteins [23], [38]. Indeed, it was recently suggested that Munc13-1 can reduce the energy barrier for SNARE-complex formation [24], and stimulates both opening and closing of syntaxin [22] implying catalytic action. Furthermore, Munc13/CAPS are Ca^2+^-dependent proteins known to affect vesicle priming [38]–[42]. Reinterpreting these proteins as catalysts for vesicle priming means that they have to be reclassified as enzymes, which might appear provocative or unusual. But it is not hard to see how proteins interacting with the SNAREs might provide an alternative pathway for assembly (and disassembly), by providing a surface stabilizing an intermediate conformation, and such mechanisms are frequently discussed in the literature. Opening and closing of syntaxin might well be stimulated by the action of a catalyst, which stabilizes an intermediate configuration. Another catalyzed step could be the initial formation of the ternary SNARE-complex, which involves the interaction of a very short, transiently formed alpha-helix in synaptobrevin with the SNAP-25:syntaxin dimer [43], [44]. Stabilization of the alpha-helix is a candidate mechanism for the catalysis of vesicle priming.

It is not necessary for the validity of our model that a protein can be found that only acts catalytically without inducing pool size effects. Instead, the catalyst may be a molecule on the plasma membrane, the association to which is necessary for priming, but which is sped up by Ca^2+^. But – unlike our simple assumption of sufficient abundance – its number may be limited, thus limiting the number of RRP vesicles. This might play a role in those cell types (e.g. neurons) where the RRP size appears to be limited by a fixed number of ‘release sites’.

### Implications for release in the syt-1 null

We introduced a specific interpretation of the release sensor, as a clamp that can be lifted by Ca^2+^ (Fig. 1). This idea aligns with recent findings and ideas that the fusion trigger consists of syt-1 and complexin in combination [27], [45], [46], even though those proteins might have additional upstream functions as well [36], [47]. Complexin and syt-1 appear to arrest SNARE-complex assembly, effectively setting up an energy barrier for fusion, which is removed when Ca^2+^ binds to syt-1. In order to explain the syt-1 null phenotype we made two separate assumptions. In the first, we assumed that the fusion barrier was removed. This caused depletion of the RRP and effectively converted the NRP into a releasable pool, because the downstream barrier was removed. Fusion in this ‘barrier-less’ model resulted in a ‘slow burst’ followed by a normal sustained component, which qualitatively matches data from syt-1 null chromaffin cells. This follows immediately from the model itself, since fusion from the NRP is the source of the slow burst in the wildtype case. In the other simulation of the syt-1 null, we assumed that syt-1 was responsible for stabilizing vesicles in the RRP, and in its absence the RRP would be nearly empty. As a result, the slow burst of release dominates after Ca^2+^ uncaging. The two models for the syt-1 null are distinguishable based on the size of the slow burst, which is larger in the second model, and the frequency of spontaneous release, which is increased in the first and decreased in the second case. The larger slow burst in the second model fits somewhat better with published data from the syt1 null [17], [31], [36], however it should be noted that syt-1 most likely has several functions in secretion [47], which could cause secondary changes in release amplitude. Therefore, we conclude that both models qualitatively account for the syt-1 null data.

In both versions of our syt-1 null model, the ‘slow Ca^2+^ sensor’ driving release in the syt-1 null is the priming catalyst, which is arranged upstream of RRP and moonlights as a Ca^2+^ sensor for release when the NRP-to-RRP transition becomes rate limiting, as it has previously been suggested [48]. This introduces a specific interpretation of the syt-1 null. In most cases, the slow release component has been assumed to either originate from a separate releasable pool (the SRP in the case of the chromaffin cell), or from an alternative sensor, which only gains access to the release machinery in the absence of syt-1. Our interpretation would explain why the ‘slow Ca^2+^ sensor’ for release – though heavily searched for – thus far could not be identified: because it is also important for fast release and the two sensors are not additive. Investigations of syt-7, which has slower Ca^2+^ (un-)binding kinetics than syt-1 [49] indeed showed that syt-7 also affected fast release in chromaffin cells [50] while its deletion in central neurons was apparently without effect [51]. Thus syt-7 does not seem to fit the description of a parallel sensor in the chromaffin cell, but the situation might be different in some neurons, including the zebrafish neuromuscular junction [52]. A recent study implicated Doc2α in asynchronous release in central neurons, but it cannot be the slow Ca^2+^ sensor itself, because asynchronous release was still Ca^2+^-dependent when Doc2α was mutated to be Ca^2+^-insensitive [53]. Thus, a testable prediction of our model is that the elusive secondary sensor should be found among Ca^2+^-dependent proteins driving priming. The alternative hypothesis of another syt-like molecule, taking the place of syt-1 or syt-2 in their absence, is also possible, but additional Ca^2+^-dependent priming reactions need to be assumed to account for the enhanced recovery of the RRP during elevated Ca^2+^ (Fig. 4D, Fig. S3), which in our model are both direct consequences of the priming catalyst.

### Conclusion: Parallel or sequential models?

Our work documents that a sequential model can describe salient wildtype and mutant phenotypes in adrenal chromaffin cells. By this, we do not mean to imply that parallel models are inherently wrong, or even unlikely, and future experiments might revive the need for such models. However, at a point in time where it seems that molecular manipulations and experiments more often than not lead to the suggestion of separate vesicle pools or sensors, we think it is important to point out that small changes in the assumptions behind sequential models might lead to similar phenotypes. Specifically, we have shown that the assumptions of catalytic action of the priming machinery, and stabilization of the RRP by syt-1, remove the necessity of assuming two fusogenic vesicle pools in the adrenal chromaffin cell. It is of note that recent experiments in cultured hippocampal neurons and in the Calyx of Held synapse led to the conclusion that the vesicles fusing during the slow phase of release are recruited to the fastest releasable pool [54], [55], in agreement with our model.

When considering neurons, other factors will contribute to kinetic diversity of release. Chief amongst these is the variable distance between RRP vesicles and Ca^2+^ channels, which might lead to kinetically distinct release phases [56]. The experiments we analyzed here were mostly performed using a spatially homogeneous Ca^2+^ signal (uncaging), which makes it easy to derive pool behavior. Future efforts will be needed to understand whether a sequential model when combined with spatial heterogeneity has explanatory power in the world of neurons.

## Materials and Methods

### Kinetic release model for adrenal chromaffin cells

To model the release from chromaffin cells we set up the reaction rate equations, which consist of a set of ordinary differential equations describing the temporal change in the population of each state (see below). We followed the suggestion by Thomas Voets [16] to simplify the scheme by assuming that the size of the Depot is so large, that it does not effectively decrease by the release (infinite Depot). We used the same parameters determined by Thomas Voets to describe the Ca^2+^ sensitivity of vesicle supply from the Depot. We also used the parameters of the release sensor found for the RRP vesicles (three site binding model [16]) to describe the release from the RRP state in our model. The full secretion model is shown in Fig. 2A and the values of all parameters can be found in Table 1. The steady state values were solved analytically under the assumption of constant NRP, RRP, RRPCa, RRPCa_2_ and RRPCa_3_ (and Depot) sizes at rest, where RRPCa_n_ represents RRP vesicles with *n* Ca^2+^ bound.

We suggest that the transition between the NRP and RRP states is catalyzed by a Ca^2+^-dependent catalyst. Therefore, the rate constants *k_2_* and *k_−2_* are functions of the Ca^2+^ concentration. A catalyst acts by increasing the rate of a reaction through the lowering of the activation energy barrier (Fig. 1). Yet a catalyst does not change the equilibrium of a reaction as it accelerates both the forward and the reverse rates simultaneously. Both reactions also occur without catalysis, but with low rates (*k_20_* and *k_−20_*). We suggest that the overall rate constants *k_2_* and *k_−2_* are functions of the un-catalyzed (Ca^2+^-independent) rate constants *k_20_* and *k_−20_* and functions of the faster, catalyzed (Ca^2+^-dependent) reaction rates *k_2cat_* and *k_−2cat_*. The relative contribution of the catalyzed rate in turn depends on the probability (*g(*Ca^2+^ *)*) of the catalyst to have bound the required number of Ca^2+^ ions in order to become activated. For simplicity, we assumed that the supply of catalyst is not limited (this could for instance mean that each vesicle bears a catalyst). The overall rate constants *k_2_* and *k_−2_* take the following form:
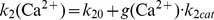
(1.1)

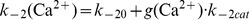
(1.2)We assume instantaneous binding of Ca^2+^ to the catalyst, so that the catalyst is in equilibrium with Ca^2+^ at all times. In general, a catalyst might bind *n* Ca^2+^ ions in order to be activated:
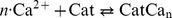
The overall dissociation constant is given by:
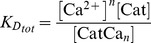
(1.3)For an individual Ca^2+^-binding step:
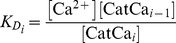
(1.4)The fraction of activated (bearing the correct number *n* of Ca^2+^ ions) to total catalyst (*g*) is
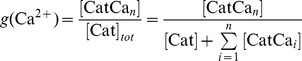
(1.5)Experimentally, the relative concentration of the active catalyst is difficult to access, whereas the Ca^2+^-levels are well defined. *g(*Ca^2+^ *)* can be rearranged:
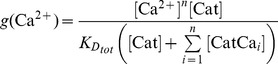
(1.6)

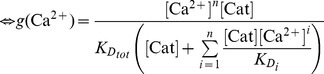
(1.7)

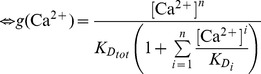
(1.8)For simplicity, it will be assumed that *n* Ca^2+^ can bind independently and with identical dissociation constants:
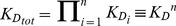
(1.9)Then *g(*Ca^2*+*^ *)* can be further simplified:
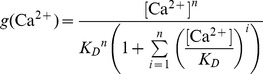
(1.10)

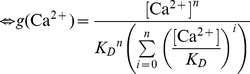
(1.11)In most of our work we assumed the simplest case of a Ca^2+^-cooperativity of one (*n* = 1). Then equation (1.11) takes the simplified form:
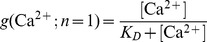
(1.12)Since a catalyst does not interfere with the free energy of RRP and NRP (same in the presence and absence of Ca^2+^), the following relationship holds true:
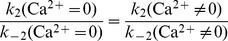
(1.13)which implies that
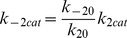
(1.14)Thus, it is sufficient to have information about the relative sizes of the NRP and RRP at equilibrium (depend mostly on the *k_20_* to *k_−20_* ratio), the uncatalyzed re-supply rate of vesicles (*k_20_*) and the asymptotic rate of NRP-to-RRP conversion at high Ca^2+^-levels to calculate all four rates.

### Estimation of parameters

We estimated the K_D_ and the *k_2cat_* values of the catalyst from the previous study by Thomas Voets (data points in Fig. 3A [16]). The *k_2cat_* corresponds to the asymptotic rate of the slow component at high Ca^2+^ concentrations. As can be seen from the data points in Fig. 3A, this corresponds roughly to a rate constant of around 20 s^−1^. According to equation (1.12), the K_D_ for a cooperativity of *n* = 1 corresponds to the Ca^2+^ concentration, where *g(*Ca^2+^ *)* = 0.5 and the slow component has reached 50% of its asymptotic rate (a value of *k_20_*+0.5*k_2cat_, where we can assume that *k_20_* is negligible). As can be seen from the data points in Fig. 3A, this corresponds to a value of roughly 100 µM. Now, *k_20_* was calculated by using equation (1.1) and investing the observation that at resting Ca^2+^ concentrations (500 nM), the rate *k_2_* is equal to 0.12 s^−1^ [32]. Finally, *k_−20_* and *k_−2cat_* were calculated using equations (1.2) and (1.14) by investing the observations that *k_−2_* at resting Ca^2+^ concentration is 0.1 s^−1^ and at steady state the *k_2_*/*k_−2_* ratio is 1.2/1 [32]. All parameters can be found in Table 1.

### Numerical integration of differential equations

The kinetic equations for the model presented in Fig. 2A take the following form:
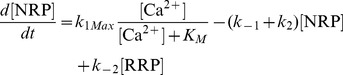
(2.1)

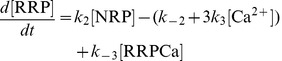
(2.2)

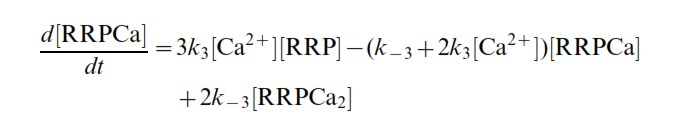
(2.3)

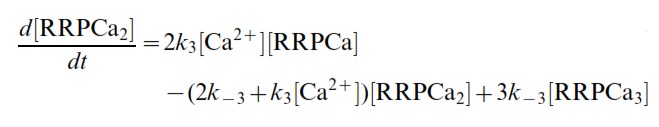
(2.4)

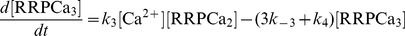
(2.5)

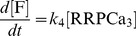
(2.6)where *k_1Max_* = 55 fF/s is a constant because of the assumption of constant Depot size [16]. The system of differential equations was integrated numerically using a fifth order Runge Kutta method with Cash-Karp coefficients and adaptive step size. For this purpose, we used a custom made macro in IGOR Pro (version 6.22A, WaveMetrics Inc.). The procedure of Runge Kutta was adapted from Numerical Recipes [57].

### Kinetic analysis

The sizes and time constants of the fast and slow release components were determined by fitting a sum of exponentials to 5 s of simulated cumulative release. The sustained release that is typically observed after the burst phase of secretion was approximated by a line. The fit function used had the following form:
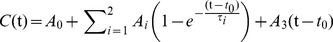
(3.1)
*A_0_* was fixed to the baseline value immediately before the onset of the Ca^2+^ step and *t_0_* was set to the inflection point of the cumulative release after stimulus onset. All other parameters were free. *A_1_* and *τ_1_* correspond to the amplitude and time constant of the fast, and *A_2_* and *τ_2_* to the size and time constant of the slow burst component, respectively. *A_3_* is the slope of the sustained component and t is time. The rate constants displayed in Fig. 3A are the inverse of the time constants.

### Refilling after depletion of the fast component in chromaffin cells

The temporal change of the two kinetic components during recovery of the fast component of release was characterized and compared to the experimental values presented in [32]. In the experiments, Voets and colleagues used a paradigm of voltage depolarizations that selectively depleted the fast component. Subsequently, the sizes of both the fast and the slow component were probed by a flash experiment (which releases both components) at varying inter-stimulus intervals [32]. Unfortunately, the precise Ca^2+^ signal at the site of the vesicle in response to the depolarization is not known. In order to test whether our model in principle would allow for a selective depletion of the fast- without major depletion of the slow component, we resorted to a simplified stimulus and probed the effect of a Ca^2+^ step from 0.5 µM to 25 µM on the NR and R states. In order to mimic the experimental conditions of the previous study we were looking for a point in time after onset of the stimulus, where the majority of fast vesicles were depleted, whereas the majority of the slow component's amplitude was still left intact, which was satisfied at the condition shown in the top panel of Figure 3D. Ca^2+^ was then stepped back to resting (0.5 µM) levels and the system was allowed to re-equilibrate for various inter-stimulus intervals before a second step to 25 µM Ca^2+^ was applied. Then, the sizes and time constants of the two components were again obtained by the fitting of exponentials (see above).

### Model comparison

For the comparison between the PPM and the SPM, the data presented in [25] were evaluated. We made use of the fact that that pre- and post-flash Ca^2+^ levels were similar in all conditions, allowing us to solve the model's differential equations under the assumption of constant *k_2_*, *k_−2_* and *k_3_* (Fig. 2A). Therefore these values are merely functions of the genetic alteration. Since we are only interested in the kinetics of burst release, we further disregarded refilling, arriving at the simple model:
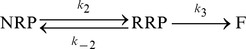
The kinetic equations can be written in matrix form:
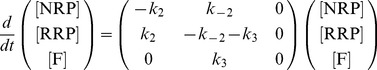
(4.1)Only the fused vesicles (*F*) contribute to the cell's increase in membrane capacitance (*C_M_*) during secretion.

To arrive at the initial conditions we assumed a fixed amount of vesicles in the burst phase (the sum of [NRP] and [RRP]), *V_tot_*. We further assumed that the NRP and RRP states are in equilibrium and that the fusion rate is essentially zero (*k_3_* = 0) before stimulation, such that:
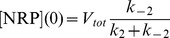
(4.2)

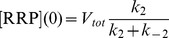
(4.3)

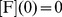
(4.4)After solving the differential equations analytically one can express the cellular capacitance change (*ΔC_M_*, which is equal to F(t) when calculating in capacitance units) in terms of the kinetic rate constants *k_2_*, *k_−2_* and *k_3_* (same rate constants as depicted in Fig. 1):
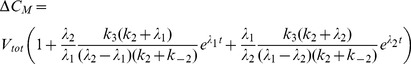
(4.5)with
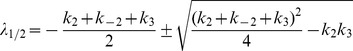
(4.6)Accordingly, the size of the fast and slow release components and their time constants are:
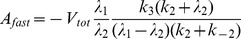
(4.7)

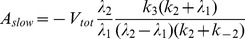
(4.8)

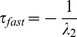
(4.9)

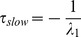
(4.10)In Figure 5C, the equation (4.5) was used to fit the total burst (1^st^ second) of release from chromaffin cells expressing wildtype and mutant synaptobrevin-2, using a custom made macro in Igor Pro 6.22A (Wave Metrics). The onset of the capacitance increase was determined manually.

The data of the measured time constants and release amplitudes following mutation of synaptobrevin-2 shown in Figure 5 were taken from Table S1 in [25]. The lines in Figure 5 represent model predictions with constant *k_2_* and *k_−2_* while varying *k_3_*. The values of *k_2_* and *k_−2_* were determined by a global fit of the model to the data sets shown in Figure 5. The behavior of the Parallel Pool Model was probed by simulating responses to Ca^2+^ uncaging (from 0.5 µM to 25 µM) for 5 s while varying the fusion rate (*k_RRP_* = *1/τ_RRP_*) in the following model:
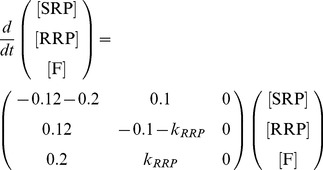
(4.11)The cumulative release was subsequently fit using the above routine. The starting values were chosen to be [*RRP*]_0_ = 61.3 fF and [*SRP*]_0_ = 52.3 fF.

## Supporting Information

Figure S1 **Fit of experimental data from chromaffin cells with cooperativity 2 for the calcium catalyst.** Same data as in Fig. 3, from [16] and [32], and model predictions of the SPM (solid lines) with the assumption that the Ca^2+^- catalyst for vesicle priming has cooperativity 2 for Ca^2+^. **A.** The rate constants for fast and slow burst release. The model with cooperativity 2 displays more saturation in the slow rate constant at high [Ca^2+^]i **B.** The sum of the fast and slow component ( = total burst, black, left hand side), or the fraction of fast- to total burst release (blue, right hand side) are fairly insensitive to post-flash Ca^2+^ [16]. The model with cooperativity 2 results in a stronger calcium-dependence of the fast component. **C.** The amplitudes of the fast- and slow release components are a bell-shaped function of the resting Ca^2+^-levels (data points from [16]). This aspect is fitted very well by the model with cooperativity 2. **D.** Following selective depletion of the fast component, its recovery is slow and at the cost of the slow component (data points from [32]). This is well fitted by the model with cooperativity 2. (See Table S1 for parameters).
(EPS)

Figure S2 **Under strong stimulation, the recovery of the fast component is sped up in the SPM, but the slow component recovers even faster.** Same experiment and simulation as in Figure 4D, but also showing the recovery of the slow component in the SPM. Points represent experimental data, solid lines represent model simulations of the fast (blue) and the slow (red) release component.
(EPS)

Figure S3 **Evaluation of an extended model based on the PPM, but with the incorporated Ca^2+^-dependent catalyst from the SPM to speed SRP-to-RRP interconversion (PPM+cat).** Same data as in Figs. 3 and 4, from [16] and [32], and model predictions of the PPM+cat (solid lines). **A.** The top panel shows the full model based on the PPM with the additional Ca^2+^-dependent rate constants, modeled as a catalyst on the SRP-to-RRP reaction (green k_2_/k_−2_, compare to Fig. 2A and 2B). Slight adjustments of the reaction rates from the SPM-catalyst were necessary (see Table S2). The bottom panel shows the rate constants for fast (blue) and slow (red) release. **B.** The sum of the fast and slow burst ( = total burst, black, left hand side), or the fraction of fast- to total burst release (blue, right hand side) are fairly insensitive to post-flash Ca^2+^ [16]. The PPM+cat model accounts well for the experimental data. **C.** The amplitudes of the fast- (blue) and slow (red) burst components are a bell-shaped function of the resting Ca^2+^-levels (data points from [16]) and well described by the PPM+cat. **D.** Following the incorporation of the Ca^2+^-dependent catalyst, also the PPM can account for a faster recovery of the fast component following strong stimulation. Same data as in Figure 4D. PPM+cat (blue solid line) mimics the experimental data (data points, same as in Fig. 4D) more closely than the PPM alone (dashed line, same as in Fig. 4D).
(EPS)

Table S1 **Model parameters for the Sequential Pool Model (SPM) with cooperativity 2 for the catalyst (See Fig. S1 for fits).**
(DOC)

Table S2 **Model parameters for Parallel Pool Model incorporating a catalyst (PPM+cat; see Fig. S3 for fits).**
(DOC)

## References

[1] Fatt P, Katz B (1952) Spontaneous subthreshold activity at motor nerve endings. J Physiol 117: 109–128. 14946732 PMC1392564

[2] Dodge FA Jr, Rahamimoff R (1967) On the relationship between calcium concentration and the amplitude of the end-plate potential. J Physiol 189: 90P–92P. 6034150

[3] Schneggenburger R, Neher E (2000) Intracellular calcium dependence of transmitter release rates at a fast central synapse. Nature 406: 889–893. 10972290 10.1038/35022702

[4] Bollmann JH, Sakmann B, Borst JG (2000) Calcium sensitivity of glutamate release in a calyx-type terminal. Science 289: 953–957. 10937999 10.1126/science.289.5481.953

[5] Lou X, Scheuss V, Schneggenburger R (2005) Allosteric modulation of the presynaptic Ca2+ sensor for vesicle fusion. Nature 435: 497–501. 15917809 10.1038/nature03568

[6] Heinemann C, Chow RH, Neher E, Zucker RS (1994) Kinetics of the secretory response in bovine chromaffin cells following flash photolysis of caged Ca2+. Biophys J 67: 2546–2557. 7696493 10.1016/S0006-3495(94)80744-1 PMC1225640

[7] Dittman JS, Regehr WG (1998) Calcium dependence and recovery kinetics of presynaptic depression at the climbing fiber to Purkinje cell synapse. J Neurosci 18: 6147–6162. 9698309 10.1523/JNEUROSCI.18-16-06147.1998 PMC6793194

[8] Hosoi N, Sakaba T, Neher E (2007) Quantitative analysis of calcium-dependent vesicle recruitment and its functional role at the calyx of Held synapse. J Neurosci 27: 14286–14298. 18160636 10.1523/JNEUROSCI.4122-07.2007 PMC6673456

[9] von Ruden L, Neher E (1993) A Ca-dependent early step in the release of catecholamines from adrenal chromaffin cells. Science 262: 1061–1065. 8235626 10.1126/science.8235626

[10] Neher E, Sakaba T (2008) Multiple roles of calcium ions in the regulation of neurotransmitter release. Neuron 59: 861–872. 18817727 10.1016/j.neuron.2008.08.019

[11] Heinemann C, von Ruden L, Chow RH, Neher E (1993) A two-step model of secretion control in neuroendocrine cells. Pflugers Arch 424: 105–112. 8414901 10.1007/BF00374600

[12] Weis S, Schneggenburger R, Neher E (1999) Properties of a model of Ca++-dependent vesicle pool dynamics and short term synaptic depression. Biophys J 77: 2418–2429. 10545345 10.1016/S0006-3495(99)77079-7 PMC1300519

[13] Trommershauser J, Schneggenburger R, Zippelius A, Neher E (2003) Heterogeneous presynaptic release probabilities: functional relevance for short-term plasticity. Biophys J 84: 1563–1579. 12609861 10.1016/S0006-3495(03)74967-4 PMC1302728

[14] Pan B, Zucker RS (2009) A general model of synaptic transmission and short-term plasticity. Neuron 62: 539–554. 19477155 10.1016/j.neuron.2009.03.025 PMC3035647

[15] Stevens DR, Schirra C, Becherer U, Rettig J (2011) Vesicle pools: lessons from adrenal chromaffin cells. Front Synaptic Neurosci 3: 2. 21423410 10.3389/fnsyn.2011.00002 PMC3059608

[16] Voets T (2000) Dissection of three Ca2+-dependent steps leading to secretion in chromaffin cells from mouse adrenal slices. Neuron 28: 537–545. 11144362 10.1016/s0896-6273(00)00131-8

[17] Voets T, Moser T, Lund PE, Chow RH, Geppert M, et al (2001) Intracellular calcium dependence of large dense-core vesicle exocytosis in the absence of synaptotagmin I. Proceedings of the National Academy of Sciences of the United States of America 98: 11680–11685. 11562488 10.1073/pnas.201398798 PMC58789

[18] Geppert M, Goda Y, Hammer RE, Li C, Rosahl TW, et al (1994) Synaptotagmin I: a major Ca2+ sensor for transmitter release at a central synapse. Cell 79: 717–727. 7954835 10.1016/0092-8674(94)90556-8

[19] Sun J, Pang ZP, Qin D, Fahim AT, Adachi R, et al (2007) A dual-Ca2+-sensor model for neurotransmitter release in a central synapse. Nature 450: 676–682. 18046404 10.1038/nature06308 PMC3536472

[20] Nadkarni S, Bartol TM, Sejnowski TJ, Levine H (2010) Modelling vesicular release at hippocampal synapses. PLoS computational biology 6: e1000983. 21085682 10.1371/journal.pcbi.1000983 PMC2978677

[21] Xu J, Mashimo T, Sudhof TC (2007) Synaptotagmin-1, -2, and -9: Ca(2+) sensors for fast release that specify distinct presynaptic properties in subsets of neurons. Neuron 54: 567–581. 17521570 10.1016/j.neuron.2007.05.004

[22] Ma C, Su L, Seven AB, Xu Y, Rizo J (2013) Reconstitution of the vital functions of Munc18 and Munc13 in neurotransmitter release. Science 339: 421–425. 23258414 10.1126/science.1230473 PMC3733786

[23] Shin OH, Lu J, Rhee JS, Tomchick DR, Pang ZP, et al (2010) Munc13 C2B domain is an activity-dependent Ca2+ regulator of synaptic exocytosis. Nat Struct Mol Biol 17: 280–288. 20154707 10.1038/nsmb.1758 PMC2916016

[24] Ma C, Li W, Xu Y, Rizo J (2011) Munc13 mediates the transition from the closed syntaxin-Munc18 complex to the SNARE complex. Nature structural & molecular biology 18: 542–549. 10.1038/nsmb.2047 PMC3087822 21499244

[25] Walter AM, Wiederhold K, Bruns D, Fasshauer D, Sorensen JB (2010) Synaptobrevin N-terminally bound to syntaxin-SNAP-25 defines the primed vesicle state in regulated exocytosis. J Cell Biol 188: 401–413. 20142423 10.1083/jcb.200907018 PMC2819690

[26] Giraudo CG, Eng WS, Melia TJ, Rothman JE (2006) A clamping mechanism involved in SNARE-dependent exocytosis. Science 313: 676–680. 16794037 10.1126/science.1129450

[27] Tang J, Maximov A, Shin OH, Dai H, Rizo J, et al (2006) A complexin/synaptotagmin 1 switch controls fast synaptic vesicle exocytosis. Cell 126: 1175–1187. 16990140 10.1016/j.cell.2006.08.030

[28] Chicka MC, Hui E, Liu H, Chapman ER (2008) Synaptotagmin arrests the SNARE complex before triggering fast, efficient membrane fusion in response to Ca2+. Nat Struct Mol Biol 15: 827–835. 18622390 10.1038/nsmb.1463 PMC2570314

[29] Yang X, Kaeser-Woo YJ, Pang ZP, Xu W, Sudhof TC (2010) Complexin clamps asynchronous release by blocking a secondary Ca(2+) sensor via its accessory alpha helix. Neuron 68: 907–920. 21145004 10.1016/j.neuron.2010.11.001 PMC3050570

[30] Kochubey O, Schneggenburger R (2011) Synaptotagmin increases the dynamic range of synapses by driving ca(2+)-evoked release and by clamping a near-linear remaining ca(2+) sensor. Neuron 69: 736–748. 21338883 10.1016/j.neuron.2011.01.013

[31] Nagy G, Kim JH, Pang ZP, Matti U, Rettig J, et al (2006) Different effects on fast exocytosis induced by synaptotagmin 1 and 2 isoforms and abundance but not by phosphorylation. J Neurosci 26: 632–643. 16407561 10.1523/JNEUROSCI.2589-05.2006 PMC6674391

[32] Voets T, Neher E, Moser T (1999) Mechanisms underlying phasic and sustained secretion in chromaffin cells from mouse adrenal slices. Neuron 23: 607–615. 10433271 10.1016/s0896-6273(00)80812-0

[33] Sorensen JB (2004) Formation, stabilisation and fusion of the readily releasable pool of secretory vesicles. Pflugers Archiv : European journal of physiology 448: 347–362. 14997396 10.1007/s00424-004-1247-8

[34] Pinheiro PS, De Wit H, Walter AM, Groffen AJ, Verhage M, et al (2013) Doc2b Synchronizes Secretion from Chromaffin Cells by stimulating fast and Inhibiting Sustained Release. J Neurosci 33: 16459–16470. 24133251 10.1523/JNEUROSCI.2656-13.2013 PMC6618527

[35] Sakaba T, Neher E (2001) Calmodulin mediates rapid recruitment of fast-releasing synaptic vesicles at a calyx-type synapse. Neuron 32: 1119–1131. 11754842 10.1016/s0896-6273(01)00543-8

[36] de Wit H, Walter AM, Milosevic I, Gulyas-Kovacs A, Riedel D, et al (2009) Synaptotagmin-1 docks secretory vesicles to syntaxin-1/SNAP-25 acceptor complexes. Cell 138: 935–946. 19716167 10.1016/j.cell.2009.07.027

[37] Sorensen JB, Wiederhold K, Muller EM, Milosevic I, Nagy G, et al (2006) Sequential N- to C-terminal SNARE complex assembly drives priming and fusion of secretory vesicles. The EMBO journal 25: 955–966. 16498411 10.1038/sj.emboj.7601003 PMC1409717

[38] Liu Y, Schirra C, Edelmann L, Matti U, Rhee J, et al (2010) Two distinct secretory vesicle-priming steps in adrenal chromaffin cells. J Cell Biol 190: 1067–1077. 20855507 10.1083/jcb.201001164 PMC3101601

[39] Junge HJ, Rhee JS, Jahn O, Varoqueaux F, Spiess J, et al (2004) Calmodulin and Munc13 form a Ca2+ sensor/effector complex that controls short-term synaptic plasticity. Cell 118: 389–401. 15294163 10.1016/j.cell.2004.06.029

[40] Lipstein N, Schaks S, Dimova K, Kalkhof S, Ihling C, et al (2012) Nonconserved Ca(2+)/calmodulin binding sites in Munc13s differentially control synaptic short-term plasticity. Mol Cell Biol 32: 4628–4641. 22966208 10.1128/MCB.00933-12 PMC3486184

[41] Ann K, Kowalchyk JA, Loyet KM, Martin TF (1997) Novel Ca2+-binding protein (CAPS) related to UNC-31 required for Ca2+-activated exocytosis. J Biol Chem 272: 19637–19640. 9289490 10.1074/jbc.272.32.19637

[42] Liu Y, Schirra C, Stevens DR, Matti U, Speidel D, et al (2008) CAPS facilitates filling of the rapidly releasable pool of large dense-core vesicles. The Journal of neuroscience : the official journal of the Society for Neuroscience 28: 5594–5601. 18495893 10.1523/JNEUROSCI.5672-07.2008 PMC6670619

[43] Ellena JF, Liang B, Wiktor M, Stein A, Cafiso DS, et al (2009) Dynamic structure of lipid-bound synaptobrevin suggests a nucleation-propagation mechanism for trans-SNARE complex formation. Proc Natl Acad Sci U S A 106: 20306–20311. 19918058 10.1073/pnas.0908317106 PMC2787132

[44] Wiederhold K, Kloepper TH, Walter AM, Stein A, Kienle N, et al (2010) A coiled coil trigger site is essential for rapid binding of synaptobrevin to the SNARE acceptor complex. J Biol Chem 285: 21549–21559. 20406821 10.1074/jbc.M110.105148 PMC2898431

[45] Krishnakumar SS, Radoff DT, Kummel D, Giraudo CG, Li F, et al (2011) A conformational switch in complexin is required for synaptotagmin to trigger synaptic fusion. Nature structural & molecular biology 18: 934–940. 10.1038/nsmb.2103 PMC3668341 21785412

[46] Malsam J, Parisotto D, Bharat TA, Scheutzow A, Krause JM, et al (2012) Complexin arrests a pool of docked vesicles for fast Ca(2+)-dependent release. The EMBO journal 31: 3270–3281. 22705946 10.1038/emboj.2012.164 PMC3411073

[47] Wang Z, Liu H, Gu Y, Chapman ER (2011) Reconstituted synaptotagmin I mediates vesicle docking, priming, and fusion. The Journal of cell biology 195: 1159–1170. 22184197 10.1083/jcb.201104079 PMC3246889

[48] Pang ZP, Sun J, Rizo J, Maximov A, Sudhof TC (2006) Genetic analysis of synaptotagmin 2 in spontaneous and Ca2+-triggered neurotransmitter release. The EMBO journal 25: 2039–2050. 16642042 10.1038/sj.emboj.7601103 PMC1462977

[49] Hui E, Bai J, Wang P, Sugimori M, Llinas RR, et al (2005) Three distinct kinetic groupings of the synaptotagmin family: candidate sensors for rapid and delayed exocytosis. Proceedings of the National Academy of Sciences of the United States of America 102: 5210–5214. 15793006 10.1073/pnas.0500941102 PMC556003

[50] Schonn JS, Maximov A, Lao Y, Sudhof TC, Sorensen JB (2008) Synaptotagmin-1 and -7 are functionally overlapping Ca2+ sensors for exocytosis in adrenal chromaffin cells. Proceedings of the National Academy of Sciences of the United States of America 105: 3998–4003. 18308932 10.1073/pnas.0712373105 PMC2268838

[51] Maximov A, Lao Y, Li H, Chen X, Rizo J, et al (2008) Genetic analysis of synaptotagmin-7 function in synaptic vesicle exocytosis. Proceedings of the National Academy of Sciences of the United States of America 105: 3986–3991. 18308933 10.1073/pnas.0712372105 PMC2268828

[52] Wen H, Linhoff MW, McGinley MJ, Li GL, Corson GM, et al (2010) Distinct roles for two synaptotagmin isoforms in synchronous and asynchronous transmitter release at zebrafish neuromuscular junction. Proceedings of the National Academy of Sciences of the United States of America 107: 13906–13911. 20643933 10.1073/pnas.1008598107 PMC2922265

[53] Yao J, Gaffaney JD, Kwon SE, Chapman ER (2011) Doc2 is a ca(2+) sensor required for asynchronous neurotransmitter release. Cell 147: 666–677. 22036572 10.1016/j.cell.2011.09.046 PMC3220409

[54] Burgalossi A, Jung S, Meyer G, Jockusch WJ, Jahn O, et al (2010) SNARE protein recycling by alphaSNAP and betaSNAP supports synaptic vesicle priming. Neuron 68: 473–487. 21040848 10.1016/j.neuron.2010.09.019

[55] Lee JS, Ho WK, Lee SH (2012) Actin-dependent rapid recruitment of reluctant synaptic vesicles into a fast-releasing vesicle pool. Proceedings of the National Academy of Sciences of the United States of America 109: E765–74 22393020 10.1073/pnas.1114072109 PMC3323990

[56] Neher E (1998) Vesicle pools and Ca2+ microdomains: new tools for understanding their roles in neurotransmitter release. Neuron 20: 389–399. 9539117 10.1016/s0896-6273(00)80983-6

[57] Press WH (2007) Numerical recipes : the art of scientific computing. Cambridge, UK ; New York: Cambridge University Press. xxi, 1235 p.

