# Supplementary material for: A Sequential Vesicle Pool Model with a Single Release Sensor and a Ca2+-Dependent Priming Catalyst Effectively Explains Ca2+-Dependent Properties of Neurosecretion
Source: PLoS Comput Biol. 2013 Dec 5;9(12):e1003362. doi: 10.1371/journal.pcbi.1003362 (PMC3854459; doi:10.1371/journal.pcbi.1003362)
Supplement: Table S1 — Model parameters for the Sequential Pool Model (SPM) with cooperativity 2 for the catalyst (See Fig. S1 for fits). (DOC) [file pcbi.1003362.s004.doc]

**Table S1.**

| **Parameter** | **Value** | **comment** |
| --- | --- | --- |
| *k_1_* | $\frac{k_{1Max}[Ca^{2+}]}{[Ca^{2+}]+K_{M}}$ |  |
| *k_1Max_* | 55 fF/s |  |
| *K_M_* | 2.3 µM |  |
| *k_-1_* | 0.05 s^-1^ |  |
| *n* | 2 | cooperativity catalyst |
| *k_2_* | $=k_{20}+g(\text{Ca}^{\text{2+}})\cdot k_{2cat}$ | see Materials and Methods |
| *k_-2_* | $=k_{-20}+g(\text{Ca}^{\text{2+}})\cdot k_{-2cat}$ | see Materials and Methods |
| *g(Ca^2+^)* | $=\frac{[\text{Ca}^{\text{2+}}]^{2}}{K_{D}^{2}+K_{D}[\text{Ca}^{\text{2+}}]+[\text{Ca}^{\text{2+}}]^{2}}$ | see Materials and Methods |
| *k_20_* | 0.103 s^-1^ | see Materials and Methods |
| *k_2cat_* | 10 s^-1^ | see Materials and Methods |
| *k_-20_* | 0.0861 s^-1^ | see Materials and Methods |
| *k_-2cat_* | $=k_{2cat}\cdot k_{-20}/k_{20}$ | see Materials and Methods |
| *K_D_* | 12 µM | see Materials and Methods |
| *k_3_* | 4.4 s^-1^μM^-1^ |  |
| *k_-3_* | 56 s^-1^ |  |
| *k_4_* | 1450 s^-1^ |  |
