# Supplementary material for: A Sequential Vesicle Pool Model with a Single Release Sensor and a Ca2+-Dependent Priming Catalyst Effectively Explains Ca2+-Dependent Properties of Neurosecretion
Source: PLoS Comput Biol. 2013 Dec 5;9(12):e1003362. doi: 10.1371/journal.pcbi.1003362 (PMC3854459; doi:10.1371/journal.pcbi.1003362)
Supplement: Table S2 — Model parameters for Parallel Pool Model incorporating a catalyst (PPM+cat; see Fig. S3 for fits). (DOC) [file pcbi.1003362.s005.doc]

**Table S2.**

| **Parameter** | **Value** | **comment** |
| --- | --- | --- |
| *k_1_* | $\frac{k_{1Max}[Ca^{2+}]}{[Ca^{2+}]+K_{M}}$ |  |
| *k_1Max_* | 55 fF/s |  |
| *K_M_* | 2.3 µM |  |
| *k_-1_* | 0.06 s^-1^ | adjusted to match pool size |
| *k_20_* | 0.0273 s^-1^ | adjusted to match RRP/SRP ratio |
| *k_2cat_* | 26 s^-1^ | adjusted to match RRP/SRP ratio |
| *k_-20_* | 0.017 s^-1^ | see Materials and Methods |
| *k_-2cat_* | $=k_{2cat}\cdot k_{-20}/k_{20}$ | see Materials and Methods |
| *K_D_* | 100 µM | see Materials and Methods |
| *n* | 1 | cooperativity catalyst |
| *k_3s_* | 0.5 s^-1^μM^-1^ |  |
| *k_-3s_* | 4 s^-1^ |  |
| *k_4s_* | 20 s^-1^ |  |
| *k_3r_* | 4.4 s^-1^μM^-1^ |  |
| *k_-3r_* | 56 s^-1^ |  |
| *k_4r_* | 1450 s^-1^ |  |
